# Mitophagy in Yeast: Decades of Research

**DOI:** 10.3390/cells10123541

**Published:** 2021-12-15

**Authors:** Ingrid Bhatia-Kissova, Nadine Camougrand

**Affiliations:** 1 Department of Biochemistry, Faculty of Natural Sciences, Comenius University, Ilkovičova 6, 84215 Bratislava, Slovakia; kissova@fns.uniba.sk; 2 CNRS, UMR 5095, 1 Rue Camille Saint-Saëns, 33077 Bordeaux, France; 3 Institut de Biochimie et de Génétique Cellulaires, Université de Bordeaux, UMR 5095, 1 Rue Camille Saint-Saëns, 33077 Bordeaux, France

**Keywords:** yeast, mitochondria, quality control, mitophagy, Atg32 protein

## Abstract

Mitophagy, the selective degradation of mitochondria by autophagy, is one of the most important mechanisms of mitochondrial quality control, and its proper functioning is essential for cellular homeostasis. In this review, we describe the most important milestones achieved during almost 2 decades of research on yeasts, which shed light on the molecular mechanisms, regulation, and role of the Atg32 receptor in this process. We analyze the role of ROS in mitophagy and discuss the physiological roles of mitophagy in unicellular organisms, such as yeast; these roles are very different from those in mammals. Additionally, we discuss some of the different tools available for studying mitophagy.

## 1. Introduction

Mitochondria are dynamic organelles that perform many functions essential to the life of eukaryotic cells. One of the main functions of the mitochondria is its role as an energy powerhouse that enables the production of ATP. This process is carried out thanks to the functioning of oxidative phosphorylations that correspond to the coupling between the respiratory chain and ATP synthase and the reoxidation of NADH (reduced nicotinamide adenine dinucleotide), which is necessary to maintain the redox potential of the cell [1,2,3]. Mitochondria are also involved in other functions, such as the synthesis of iron-sulfur centers, certain stages of β-oxidation of fatty acids, the Krebs cycle, heme biosynthesis, and the metabolism of certain amino acids and lipids. In addition to being irreplaceable for providing vital cellular functions, mitochondria are also key players in programmed cell death, or apoptosis [4]. During apoptosis, the outer mitochondrial membrane permeabilizes and, as a result, cytochrome *c* and apoptogenic factors are released into the cytosol, triggering a cascade of execution processes for cell death. In mammals, mitochondria are essential sites for the biosynthesis of steroid hormones and are closely associated with calcium homeostasis. All of these metabolic reactions are made possible by the numerous transporters present in the mitochondrial membranes, ensuring exchanges between the mitochondria and the cytosol. The mitochondria are the main intracellular source of reactive oxygen species (ROS). Under physiological conditions, superoxide ions are constantly generated in the mitochondrial respiratory chain [5,6,7,8]. These ROSs can present either beneficial effects or toxic effects for the cell [9,10]. Mitochondrial DNA, which is more vulnerable than nuclear DNA because of the absence of histones, can be a target of these ROSs, as well as proteins and lipids. Oxidative damage and oxidized biomolecules are considered to be potential contributors, or associated factors, to certain pathologies, such as neurodegenerative diseases and diabetes, or to the appearance of cancers. The build-up of ROS-related damage over the course of cells is also often seen as one of the major causes of cellular aging [11,12].

In view of the place and role of mitochondria in cell homeostasis, quality control and regulation of the turnover of these organelles are particularly important. Mitochondrial homeostasis is provided by a robust system of processes and pathways involved in rearranging the content and changing the amount of mitochondrial proteins in response to varied environments or stressful situations. Quality control of mitochondria occurs at different levels. Chaperones and proteases located in the various mitochondrial compartments, as well as the ubiquitin–proteasome system, act at the molecular level by monitoring proteins [13,14,15,16]. When the mitochondrial damage is too great, and often irreparable, it is necessary for cells to put in place another process to eliminate these failed mitochondria. In mammals, the first control is by the formation of mitochondrial derived vesicles (MDVs), 70–150 nm in diameter, composed of one or two membranes, and enriched with specific mitochondrial protein [17,18,19]. This process is enhanced especially when the mitochondria are under oxidative stress, a situation that cells often have to face [20]. At the organelle level, the second process that occurs is mitophagy. Mitophagy corresponds to the selective degradation of mitochondria by autophagy. Autophagy is one of the cellular degradation processes that is conserved in all eukaryotic organisms. It involves molecular machinery and the following specific degradation compartments: the vacuole in yeasts and plants and lysosomes in mammals. The first autophagy events were observed in the mid-twentieth century [21]. Dr. Christian De Duve was awarded the Nobel Prize in Physiology or Medicine in 1974 for having described the role of the lysosome in the degradation of cellular constituents. Several forms of autophagy have been described, but macroautophagy is considered to be the main form of autophagy. It involves creating a double-membrane structure, which further enlarges and encloses cellular material to form vesicles called autophagosomes. These structures then fuse with the lytic organelles, and their content is subsequently degraded thanks to the various enzymes present in the compartments such as lysosomes or vacuoles [22] for review. Many diseases, such as Parkinson’s, Alzheimer’s, and cancers have been linked to autophagy defects (see [23] for review). Autophagy is a nonselective process because it randomly sequesters portions of cytoplasm, including organelles. This process can become selective if it specifically targets a cellular constituent. The selective degradation of mitochondria is called mitophagy [24].

## 2. Yeast as a Study Model

Among the many species of yeast found in nature, *Saccharomyces cerevisiae*, called baker’s yeast, is a eukaryotic organism that shares many basic cellular processes with mammalian cells. For years, *S. cerevisiae* has been used as a key organism for the identification and understanding of the molecular mechanisms underlying the fundamental functions of all eukaryotic cells. It has the following advantages: it is a nonpathogenic organism, exhibits easy handling and rapid growth in the laboratory on solid or liquid media, has a small fully sequenced genome, and facilitates ease of genetic manipulation. *S. cerevisiae* can reproduce in two forms as follows: identically, by budding a daughter cell from mother yeast, or by sexual reproduction. *S. cerevisiae* is capable of following two metabolic pathways, the aerobic pathway and the anaerobic pathway. This allows it to live in diverse environments. For the aerobic route, yeast uses aerobic respiration to metabolize carbohydrates into carbon dioxide and water. For the anaerobic route, it ferments carbohydrates and produces ethanol and CO_2_. It is thus possible to orient the metabolism of this yeast according to the carbon source added to the culture medium.

It should also be noted that a small number of strains of *S. cerevisiae* are used in laboratories (i.e., S288c, YPH499, BY4243, BY4242, BY4241, X2180-A, X2180-B, CEN.PK, D273-10B, W303-1A, W303-1B, SEY6210). The S288c strain is the most popular and has been used for genome sequencing [25,26,27]. The majority of the strains descend from the S288c strain but present different mutations, which give them physiological differences [28,29]. This is the case, for example, between strains W303 and BY—the latter of which has an insertion of a transposon in the *HAP1* gene that gives it an altered mitochondrial phenotype [30]. This strain has half the number of cytochromes, which are constituents of the respiratory chain, compared with the W303 strains. The Euroscarf bank, which offers all the deletion mutants of the various viable genes, was built with the BY strains. This bank of mutants can be a tool for implementing genetic screens. 

Several tools are available to study mitophagy in yeast. The proteins addressed to the mitochondria, such as Atp9 (1-69) + DHFR (an artificial chimeric mitophagy reporter), or mitochondrial proteins of the different compartments labeled with GFP, for example Om45-GFP (outer membrane), Idp-GFP, and Idh-GFP (matrix), are used either for observations in fluorescence microscopy or in western blots to visualize green vacuoles or the appearance of the free GFP form that is more resistant to proteases. A mitochondrial chimeric protein GFP-RFP was also used to follow mitophagy and especially the last step in the vacuole.

Care must be taken in the use of these different fusion proteins because they are not degraded in the same way and at the same rate by mitophagy. Indeed, Kolitsida et al. (2019) has recently shown an unexpected dimension in protein level selectivity with different rates of protein degradation [31]. This selectivity is regulated by differential phosphorylation/dephosphorylation events. Mitophagy events could also be observed by electron microscopy and immuno-electron microscopy. The last, more quantitative technique is based on the reporter activity of a mitochondrially targeted mutant version of alkaline phosphatase Pho8 (mtPho8), described for the first time by Campbell and Thorsness [32] (for more details see next paragraph).

## 3. Mitophagy in Yeast

The study of autophagy in the yeast system began almost 30 years ago, when Dr. Yoshinori Ohsumi showed that yeast displayed a similar autophagic morphology to that observed in mammalian cells. Such substantial findings opened the door for further study of autophagy in this genetic model organism. This pilot study also showed that autophagosomes, which formed after autophagy induction prompted by nitrogen starvation in a medium with fermentative carbon source glucose, contained various cytosolic contents (including mitochondria) and indicated a random fashion of mitochondria elimination in yeast [33].

In the following years, Dr. Ohsumi and his colleagues used genetic screenings to identify many *ATG* genes involved in the mechanism and regulation of yeast autophagy. For his work on autophagy in yeast, Dr. Ohsumi was awarded the Nobel Prize in Physiology or Medicine in 2016. 9452320

A selective accumulation of mitochondria in vacuoles was reported in *yme1*Δ yeast, with mutant cells grown in a glycerol/ethanol medium favoring respiratory metabolism [32]. To assess the vacuolar-dependent degradation of mitochondria, the authors modified an in vivo test using the *PHO8* reporter system that was originally developed by Noda et al. to quantify autophagy in yeast [34]. Encoding the vacuolar alkaline phosphatase (ALP), *PHO8* is synthesized in the form of an inactive proenzyme, and its maturation occurs only after its delivery to the vacuoles, where its C-terminus is cleaved by proteinase A [35]. For their assay, the authors prepared a mutant form of *PHO8*, from which they removed the first 60 codons carrying the vacuolar import signal and replaced them with the mitochondrial-directed signal sequence from *COX IV*. Recombinant ALP was targeted into mitochondria (mtALP) in a strain-carrying disruption of the genomic alkaline phosphatase *loci*, *PHO8*, and *PHO13,* to ensure that only significant ALP activity is mtALP. Maturation of the mtALP proenzyme by proteolytic cleavage will happen only after vacuolar uptake of the mitochondria. Measurement of mtALP activity in strains bearing different mutations and under different growth or stress conditions allow evaluation of the relative rate of vacuole-dependent mitochondrial turnover. A study by Campbell and Thorsness (1998) revealed that mitochondria in *yme1*Δ mutant cells are delivered into vacuole more frequently when compared to the rate observed in wild-type strain. Yme1 is an AAA ATPase involved in the escape of mitochondrial DNA (mtDNA) from mitochondria to the nucleus in *S. cerevisiae* [32]. In cells growing on glycerol/ethanol, almost no transfer of mtDNA was detected in wild-type yeast. Conversely, in the absence of *YME1*, a huge increase in the rate of escape of mtDNA was observed. This correlated with emergence of “squeezed” mitochondria, which broke into small fragments arranged alongside invaginations at the surface of the vacuole. Hence, Yme1p was the first identified protein whose absence triggered a selective turnover of mitochondria. However, whether autophagy was involved—and what kind of autophagy it was, if so—remains unclear [32]. 

Yeast cells are equipped with multifaceted metabolism, which changes depending on the carbon source available in the medium. Fermentable sugar glucose is widely preferred and is the most commonly used in yeast biology studies. However, respiration is repressed by glucose even in the presence of oxygen (the Crabtree effect), which prevents the development of fully functional mitochondria. Thus, glucose is not the most ideal carbon source for the study of these organelles in yeast. The first study showing the selectivity of the process in the degradation of mitochondria by autophagy used cell culturing media with a strictly respiratory substrate, such as lactate, along with yeast strain suitable for the study of mitochondria, strain W303-1B [36]. Under these growing conditions, a non-fermentable carbon source is oxidized to pyruvate by two mitochondrial lactate dehydrogenases that directly transfer electrons to the mitochondrial respiratory chain. This strictly mitochondria-dependent metabolism provides an optimal differentiation of a higher number of mitochondria. The observations described in this article from 2004 marked a breakthrough in the study of yeast mitophagy because they revealed conditions in which rapid mitochondrial degradation can be observed by treating cells with rapamycin or depleting nitrogen in the medium. The authors detected mitochondrial degradation using fluorescence microscopy on cells that expressed a protein from the mitochondrial matrix labeled with GFP, or by monitoring the turnover of selected mitochondrial proteins (Cox2, Porin) by western blots. Within 2 h of treatment with rapamycin, the mitochondrial network was disrupted, and mitochondrial proteins were degraded. This degradation correlated with the appearance of a mitochondria/vacuole co-staining as GFP-labeled mitochondria were delivered into vacuoles. Mitochondrial degradation was autophagy- and vacuole-dependent because it did not occur in the autophagy-deficient *atg5*Δ mutant or in the vacuolar proteolysis-deficient *pep4*Δ mutant. Conversely, mitochondrial degradation was vastly delayed in the *uth1*Δ mutant, where the autophagic equipment is entirely functional, and vacuolar proteolysis is not affected. Thus, the mitochondrial protein Uth1p was the first described protein involved in the regulation of autophagic mitochondrial degradation [36]. However, the location of this protein was controversial. In 2006, mitochondria from *uth1*Δ mutant cells expressing *galUTH1-V5* (W303-1B background) were isolated and purified on a density sucrose gradient. Then, mitochondria were suspended in a hypo-osmotic buffer and treated, or not, with proteinase K. Afterwards, both mitochondrial membranes (inner membrane and outer membrane) were also purified. The results showed that this protein is localized in the outer membrane of the mitochondria [37]. In contrast, in 2013, in a strain BY4742, Thum et al. showed that the permeabilization of the outer mitochondrial membrane by osmotic swelling or sonication led to protease accessibility of Uth1-FLAG, confirming localization of Uth1p in the inner mitochondrial membrane [38]. In the first study, the Uth1 protein was tagged with the V5 tag at C-terminus, and the gene was under the control of the *GAL* promoter [37]. In the second study, the protein was tagged with the FLAG tag, and the corresponding gene was inserted at the locus under its own promoter [38]. The sequence of the *UTH1* gene shows the presence of two *ATG* codons, which could correspond to two translation start sites. In the second study, during in vitro experiments to follow the import of the Uth1 protein, two bands of different molecular mass were visible in the lysate. The highest band has been followed and studied. It could correspond to the longest protein (first *ATG)*. The overexpression of the protein in the first study could possibly lead to changes in the localization of the protein that show a lower molecular mass (translation from the second *ATG*). Additional experiments should be carried out to elucidate what form of the protein is actually synthesized (long form or short form), including its location and its role in the mitochondria. 

Additionally, the selectivity of mitochondria degradation by autophagy was confirmed by using electron microscopy and immuno-electron microscopy. Moreover, it was also shown in this study that the mitophagy process is dependent on core autophagic machinery—and *ATG1*, *ATG5*, *ATG8*, *ATG9*, *ATG12*, *ATG13*, *VAC8*, *VAM7* are essential for the process [39].

It was assumed that, in the absence of sufficient nutrients, mitophagy allows the cell to get rid of redundant, or possibly altered organelles, whose maintenance would demand too much energy in a given situation. In 2005, Priault et al. described for the first time the induction of autophagy as a result of mitochondrial failure to function properly in either the *fmc1*Δ mutant subjected to heat stress, or in a mutant bearing a point mutation in the *ATP2* gene growing in anaerobic conditions in fermentable glucose-containing media [40]. In these conditions, only the mitochondrial electrical (Δ*Ψ*) potential was affected because there was no change in the intracellular ATP concentration, and mitochondrial biogenesis was not affected. In these conditions, autophagy allows the elimination of defective mitochondria and eventually leads to death. Nevertheless, evidence about the selectivity of the process in this study was unclear. The induction of autophagy, occurring in the aftermath of mitochondrial damage, can be seen as a mechanism by which the cell controls the function of its organelles and allows the cells to survive. As such, this pathway is a stress-induced housekeeping mechanism involved in the general maintenance of cellular homeostasis (see [41] for review).

In 2007, Nowikovsky et al. reported that a loss of K(+)/H(+) exchange activity of the mitochondrial Mdm38 protein triggers autophagy and the degradation of mitochondria by microautophagy [42]. In this study, the authors use a novel fluorescent biosensor composed of a pH-sensitive GFP and a pH-insensitive RFP, targeted to a mitochondrial matrix, and use the FM4-64 dye to label vacuoles. The use of a different approach led to the discovery that an imbalance in mitochondrial ion homeostasis caused by the absence of Mdm38 protein leads to morphological changes of both mitochondria and vacuoles, and causes close contacts to form between these organelles [42]. The addition of nigericin, a K(+)/H(+) ionophore, reversed the phenotype of the *mdm*38Δ mutant in the W303 background. It was concluded that osmotic swelling of mitochondria provokes selective autophagic elimination of mitochondria [42]. Despite this, the electron microscopy images presented in the article did not exhibit any apparent characteristics of a selective process. Further, cells missing Mdm38p were more prone to die in the stationary phase. It is unclear whether stimulation of autophagic pathways was responsible for cell death or whether K(+)/H(+) affected the exchange flaw.

Tal et al. (2007) characterized the Aup1/Ptc6 protein, one of the seven members of the protein phosphatase 2C family in the yeast *S. cerevisiae* [43]. It is located both in the intermembrane space and in the mitochondrial matrix, and controls phosphorylation of the alpha subunit of pyruvate dehydrogenase, Pda1p. The Tal et al. study showed that the Aup1 protein is required for cell survival in the stationary phase of growth in a medium containing respiratory carbon source lactate, and is also necessary for effective mitochondrial autophagy under this condition. 

The same laboratory demonstrated that mitophagy induction during the stationary phase correlated with the onset of retrograde pathway target genes (*RTG*) in an Aup1-dependent manner, likely through the Rtg3 transcription factor [44]. To identify the specific mechanisms through which mitochondria altered by oxidative damage or dysfunctional mitochondria trigger their own degradation during the stationary phase, additional components of this pathway need to be explored. 

In 2008, the first genetic screens were performed, this time by culturing the BY4742 yeasts in media with a strictly respiratory carbon source followed by a switch in nitrogen starvation in presence of fermentative carbon source [45]. For the observation of mitophagy by fluorescence microscopy, the authors used a similar approach as was used by Kiššová et al. [36,39] by employing mitochondrial proteins tagged with GFP. Additionally, Kanki and Klionsky used a chimeric reporter containing a mitochondrial protein tagged with GFP to monitor mitophagy by western blots. This approach takes advantage of how recombinant mitochondrial protein is degraded by proteases only upon delivery to the vacuoles, releasing an intact form of free GFP that is relatively resistant to proteolytic cleavage [45]. Using this method, they screened several *ATG* mutants and found that *Atg11*, a gene that is essential only for selective autophagy, is also essential for mitophagy [45], as well as the core machinery mutants, as shown in Kiššová et al. [39]. Using the same type of screen with the Om45-GFP protein, and the visualization, or not, of fluorescence in the vacuole after several days of growth in a lactate medium, the Atg33 protein, located in the mitochondrial outer membrane, was also found to exist [46].

## 4. Key Role of the Atg32 Protein

In 2009, the first yeast mitophagy receptor was revealed from two independent genetic screens. Okamoto et al. [47] and Kanki et al. [48] used similar strategies and identified mitochondrial-anchored Atg32 protein as essential for mitophagy but not nonselective autophagy.

To identify which genes are involved in mitophagy, the first lab carried out a genome-wide visual screen for mutants that were defective in transporting mito-GFP (Atp9 (1-69) + GFP) to the vacuole during induction of mitophagy in the stationary phase [47]. They tested approximately 5150 strains with nonessential gene deletion in BY4741 background.

Among the 53 mitophagy-defective strains initially identified in their first screen, 30 mutants were already described as impaired in autophagy and/or the Cvt pathway, and 23 were not known to be involved in autophagic processes. With respect to their function, these 23 mutants represent a group of proteins involved in diverse functions, such as in lipid and mitochondrial metabolism, protein modification and degradation, and membrane trafficking. This suggests mitophagy is more than a simple degradation pathway and requires the participation of a broad variety of protein components and cellular functions. Aup1 and Uth1 proteins, which have been determined to be a requirement for mitophagy [36,39,43], were not identified as essential for selective mitochondrial elimination under their conditions. This is normal because mitophagy is only delayed in the *uth1*Δ and *aup1*Δ mutants. Among all identified mutants, a mutant with a deletion of uncharacterized *ECM37* gene (*YIL146C*) exhibited an almost complete loss of mitophagy. The gene was named Atg32 [47]. The same authors confirmed the absence of mitophagy in this mutant by fluorescent microscopy, western blots with mito GPD-mt DHFR fusion protein (Atp9 (1-69) +DHFR + GFP), and electron microscopy. They showed that Atg32 was localized in the outer mitochondrial membrane and interacted with Atg11 and Atg8 proteins [47].

At the same time, Klionsky’s lab performed a genomic screen by transforming the yeast knockout library (BY background) by a DNA fragment, allowing researchers to label the *OM45* gene at the chromosomal locus with GFP [48]. They followed mitophagy in cells grown in a medium supplemented with lactate as the sole carbon source (YPL) for several days after cells entered a stationary phase by tracking the appearance, or absence, of the GFP fluorescence inside a vacuole. From this analysis, they obtained the same strain with a deletion in the *YIL146C/ECM37* gene as Ohsumi’s lab. In their setting, the *yil146c*Δ strain was entirely impaired in mitophagy. They confirmed the data using fluorescent microscopy, western blots, and electron microscopy. They found the same outer mitochondrial membrane localization and interaction with the Atg11 protein [48].

Among all proteins playing a role in yeast mitophagy, the Atg32p is only strictly essential for the process of mitophagy in all known conditions, and it is defined as the mitochondrial receptor for mitophagy [47,48]. It is localized on the outer mitochondrial membrane, and can interact with Atg11 protein, which acts as an adapter, as well as the Atg8 protein present in the phagophore membrane. These interactions ultimately allow for the sequestration of the mitochondria in the autophagosomes to be degraded.

Up to now, Atg32p is the only known mitophagy receptor in *S. cerevisiae*. For its importance, Atg32 expression and activity are subject to precise complex regulation. At the transcription level, several repressors [49,50] and activators [51,52,53] were described to control or link with Atg32 expression. An additional mechanism for conducting mitophagy induction is Atg32 phosphorylation [54,55,56]. In addition to phosphorylation, Atg32p endures other forms of post-translational modifications, such as proteolytic processing [57,58], ubiquitination [59] and other post-translational modifications not identified yet [60]. Molecular mechanisms of mitophagy in yeast are discussed in the review proposed by Innokentev and Kanki (2021), which is a part of this Special Issue, entitled: “Mitophagy in yeast: molecular mechanism and regulation”.

## 5. An Unconventional Pathway for Degradation of Mitochondria in Yeast

The most studied and best understood mitophagy is a conventional Atg32-dependent pathway that needs a receptor for identification and sequestration of mitochondria by the autophagic structures. Hughes et al. (2016) described an unorthodox pathway for mitochondrial elimination activated in aged yeast cells undergoing vacuole-induced mitochondrial dysfunction [61]. This form of protein degradation does not require the participation of Atg32p, and it removes the specific set of mitochondrial outer and inner membrane proteins that rely on the Tom70 import pathway through a formation of mitochondrial-derived compartments (MDCs). The process of MDC formation requires the participation of the mitochondrial fission machinery and the Dnm1 protein in particular. The cargo selectivity of the MDC pathway is achieved via the importer proteins Tom70 and Tom71. After their formation, all or portions of the MDCs are released from the mitochondria to be subsequently engulfed by autophagosomes, followed by delivery to the vacuole and degradation. The researchers observed that cells that failed to form MDCs displayed a greater loss of membrane potential following disruption of vacuolar acidity [61], which suggests the formation of MDC protects mitochondria against vacuole-induced stress. Because the absence of the Dnm1 protein extends the lifespan of yeast, it is possible to speculate about whether mitochondrial fragments containing Tom70p do not contribute to cytotoxicity and/or death in aged yeast [62]. 

MDCs resemble mitochondrial-derived vesicles, an autophagy-independent mechanism of mitochondrial quality control only identified in mammalian cells [19]. The MDC pathway selectively disposes of a set of mitochondrial membrane-associated proteins. This is mechanistically distinct from autophagy-dependent systems involving receptors that allow vacuolar turnover of whole portions of mitochondria (for review see [63,64]).

**Figure 1:**
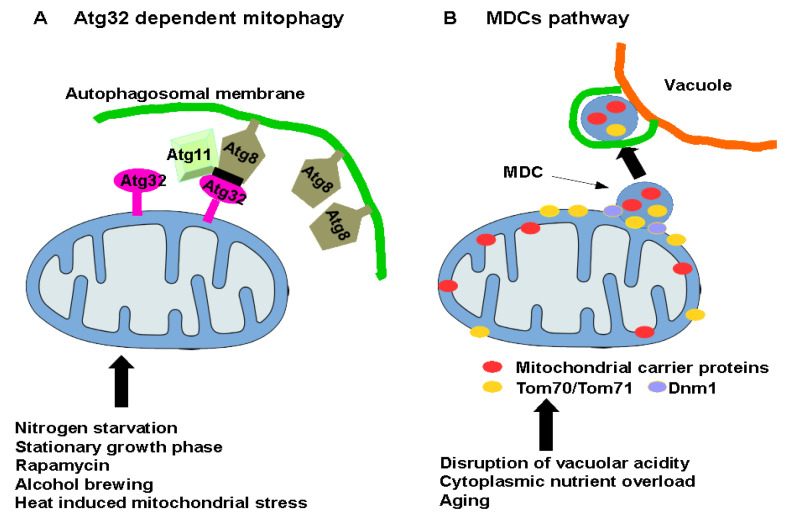
Pathways involved in selective removal of mitochondria in yeast. (**A**) Conventional mitophagy relies on receptor protein Atg32. Following the activation of mitophagy, Atg32p binds to Atg8p conjugated to phosphatidylethanolamine at PAS, and Atg11, an adaptor protein, to confer selectivity for mitophagy. (**B**) The mitochondria-derived compartments (MDC) pathway. This process selectively sequesters a group of mitochondrial membrane proteins, mainly carrier family proteins, into vesicles that bud from the mitochondria in a Dnm1- and Tom70/71-dependent way followed by elimination by autophagy.

The MDC pathway allows the destruction of a variety of mitochondrial substrates, including a dominant group of the mitochondrial transporters that mediate the exchange of nutrients and metabolites across the inner mitochondrial membrane [65]. As a result of the loss of vacuole acidity, the mitochondria are damaged, leading to the disruption of amino acid storage in the vacuolar lumen [66]. The MDC pathway could thus contribute to sequestration of mitochondrial carrier proteins in the event of excessive accumulation of nutrients in the cytosol [67] in an effort to prevent a deregulated or unsustainable influx of nutrients into the mitochondria. Alternatively, the MDC pathway may destroy the nutrient carrier proteins to adapt mitochondria’s metabolism so the cell can better cope with vacuole dysfunction [61] (Figure 1).

## 6. Induction of Mitophagy in *S. cerevisiae*

**Figure 2:**
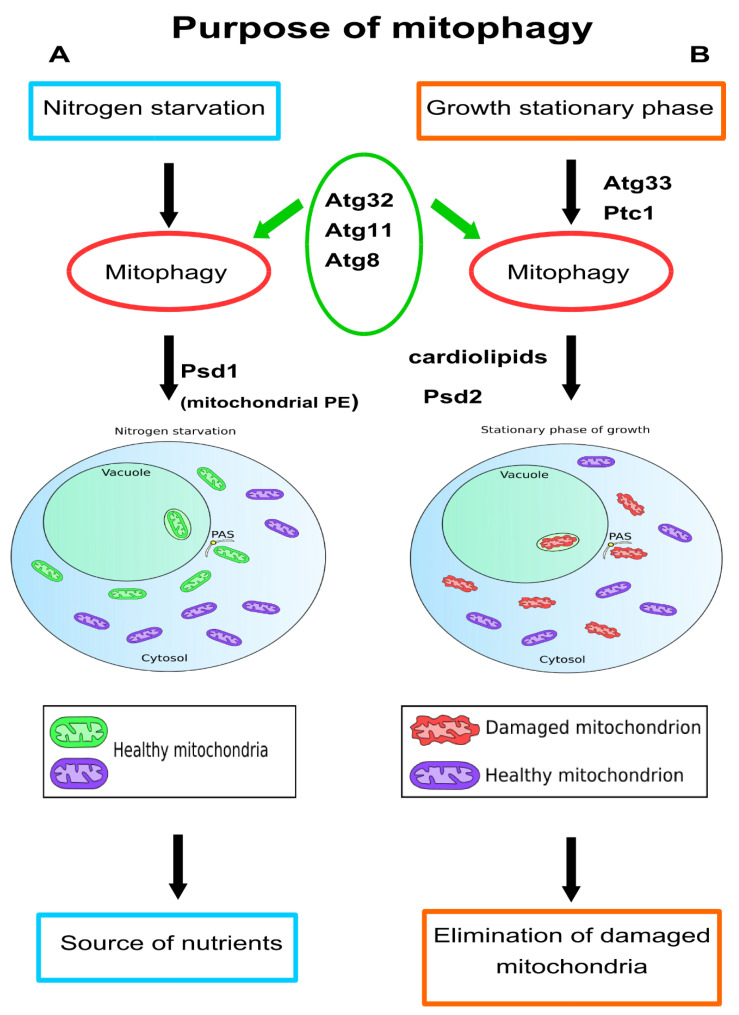
Mitophagy in yeast employs different signaling pathways in a context-dependent manner. In *S. cerevisiae*, mitophagy can be triggered by nitrogen starvation or stationary phase of growth. In each way, cells are cultured in media with respiratory carbon source and the process requires the same key proteins, such as Atg32, Atg11, and Atg8. (**A**) Cells submitted to starvation are harvested in a mid-exponential growth phase, and under these conditions most mitochondria are healthy and functional. Here, the role of mitophagy is to provide new nutrients by recycling redundant organelles to ensure cell survival in adverse conditions. (**B**) During the stationary phase of growth, mitophagy is useful to eliminate mitochondria that have been damaged or dysfunctional to maintain cellular function and homeostasis. The fact that proteins Aup1, Atg33, and Psd2 are required for the stationary phase mitophagy, but are indispensable for mitophagy induced by starvation, and conversely, that mitochondrial Psd1p is selectively required for the removal of mitochondria in nitrogen deficiency, suggests that different signaling pathways are involved in the induction of mitophagy [68].

Mitophagy in yeast *S. cerevisiae* can be triggered in several ways. In each way, it is essential that cells grow first in a medium supplemented with a non-fermentable, respiratory carbon source (e.g., lactic acid or glycerol) that promotes respiratory metabolism and allows full differentiation of the mitochondria. Cells can then be switched to a nitrogen-starvation medium supplemented with different carbon sources. In the first scenario, cells continue to starve for nitrogen in strictly respiratory conditions, in which high mitochondrial activity is further sustained and ROS continue to alter or damage biological structures. In the alternative protocol, cells are transferred to a starvation medium with a fermentative carbon source (e.g., glucose), in which budding yeast mainly obtains energy from glycolysis. Such a change leads to the degradation of redundant mitochondria to replenish depleted amino acid supplies caused by nitrogen starvation. Mitophagy is also induced by culturing yeast cells to the post logarithmic phase (2–3 days), referred to as stationary phase mitophagy. At this growth phase, energy requirements are reduced, so cells do not need to maintain excess mitochondrial mass. In this case, mitophagy is the tool by which the cell controls quality and gets rid of dysfunctional or damaged mitochondria that accumulate over time in aging cells [Figure 2].

The least used method for inducing mitophagy is treating yeast with the drug rapamycin. Mitophagy is not induced in cells that were pre-cultured in a medium with a respiratory carbon source after they have been switched into a nutrient rich medium with a fermentative carbon source such as glucose [36]. Here, the quantity of mitochondria may rapidly decrease in response to glucose repression. Yeast adapt quickly to their molecular and cellular actions/tasks in response to the quantity of glucose in a media because glucose suppresses gluconeogenesis and respiration.

Interestingly, W303-1B strain *S. cerevisiae* can degrade mitochondria selectively through the following two morphologically distinct mitophagic pathways: micromitophagy and macromitophagy. Micro- and macromitophagy are induced by adapting cells grown in strict respiratory conditions to starvation in media with a respiratory or fermentative carbon source, respectively. Micromitophagy is induced by lactate, and macromitophagy is induced by glucose [39,45]. During micromitophagy, mitochondria are directly engulfed into lytic organelles by invagination or protrusions of vacuolar membranes. In contrast, during macromitophagy, mitochondria are sequestered primarily by being included within newly formed membranes. The mitochondria containing autophagosomes then fuse with vacuole to deliver cargo. Due to these differences in membrane dynamics, micro- and macromitophagy pathways can be easily distinguished by electron microscopic observation, but our understanding of the molecular signal(s) that determine which pathway is activated or repressed in response to environmental changes is limited. Both pathways are Atg32-dependent. The Uth1 protein is essential for micromitophagy [39]. It has not yet been determined whether intracellular ATP levels play a role in determining which mitophagy pathway is induced, as described in the case of pexophagy in *P. pastoris* [69]. In addition, the absence of nutrients only marginally affected vacuolar morphology in glucose-grown cells, but caused extreme changes in morphology in lactate-grown cells [39]. It is unclear whether this alteration in vacuolar morphology is one of determinants regulating the mitophagy pathways, especially in the switching process between micro- and macromitophagy.

## 7. Are ROS the Main Culprits?

An increase in levels of intracellular ROS often correlates with damage, particularly to mitochondria. Cells grown in respiratory conditions exhibit a well-differentiated mitochondrial network. Under the conditions of physiologically induced mitophagy and autophagy by nitrogen starvation that triggers increased ROS production, mitochondrial morphology is altered, and the network is disrupted and fragmented [70]. Oxidative stress may be a crucial factor involved in the induction of mitophagy because the antioxidant N-acetylcysteine (NAC) can completely prevent mitophagy induction in all the conditions described above, without any effect on bulk autophagy [70]. However, other antioxidants such as Tiron, ascorbic acid, and resveratrol have no effect on mitophagy. What makes NAC special/different from other substances? After being converted to cysteine, NAC stimulates the synthesis of glutathione (GSH). The presence of a thiol group may help prevent or reduce moderate oxidative stress. The process of preserving the adequate concentration of GSH is pivotal for protecting cells against oxidative stress and regulating intracellular signaling [71]. This process is even more key when the cells are exposed to nutrient deficiency because the precursors needed for the synthesis of GSH are not readily available. The acetylation of the amine group in NAC provides an ideal precursor for GSH biosynthesis. Thus, under this condition, the main effect of NAC might be to supply the L-cysteine reservoir for GSH metabolism. Deffieu et al. (2009) reported a rapid decrease in GSH content within cells whose medium was depleted of nitrogen. At the same time, they showed that the addition of NAC allowed for restoration of the intracellular level of GSH and reversal of an imbalance in the redox state of cells that is illustrated by an increase in NAD(P)H/NAD(P)+ ratios [70]. A condition with impaired intracellular redox balance, not fully active mitochondria, and the presence of molecular oxygen could potentially be detrimental for cells. NAC-stimulated biosynthesis of GSH through restoring cellular NAD(P)H/NAD(P)+ ratios possibly relieves the need for mitochondria removal. 

Alternatively, glutathione can modulate the activity of a variety of proteins via *S*-glutathionylation of cysteine sulfhydryl groups. Therefore, it is possible that thiol-containing proteins targeted by glutathione could play a role in the regulation of mitophagy. There is also an unanswered question as to whether glutathione could play a role in clearing mitochondrially generated oxidants in vacuoles in the final step of mitophagy [72]. 

In parallel, it has been shown that the addition of NAC decreased Atg32p levels and mitophagy when cells were grown in a glycerol medium and reached the stationary phase [47].

Compared to what happens in mammals, in yeast, chemically induced oxidative stress (by hydroxide peroxide and Antimycin A) or loss of mitochondrial membrane potential caused by mitochondrial oxidative phosphorylation uncouplers (such as CCCP and FCCP) do not induce mitophagy in *S. cerevisiae* [36,61,73].

Intriguingly, Antimycin A, an inhibitor of cellular respiration, induced bulk autophagy in wild-type cells of *S. cerevisiae* grown in a medium with a respiratory carbon source [73]. However, treatment with Antimycin A did generate low or no autophagic activity in mutants lacking proteins that play a role in mitophagy, such as Atg11, Atg32, and Bck1 (a kinase participating in MAPK-signaling pathways that contributes to the regulation of mitophagy through the phosphorylation of the Atg32) [74]. Although Atg32 protein is necessary exclusively for mitophagy, Atg11 is needed for all forms of selective autophagy. It is particularly surprising that even if the *atg32*Δ mutant does not display any impairment in nonselective autophagy during starvation or stationary phase, it is indispensable for Antimycin-A-induced autophagy. At the same time, proteins that are involved or modulate mitophagy but are generally not essential for this process (such as Atg33, Ptc6/Aup1, and Dnm1) do not interfere with Antimycin-A-induced autophagy. These observations suggested a novel and major role that Atg32p could play in the control of autophagy as part of a cell’s response to mitochondrial alteration. In the same study, the authors showed that a level of a reduced form of cytochrome *b*, one of the components of the respiratory chain, could be the first signal in the pathway leading to the activation of autophagy in this situation. Molecules that seize the signal and direct it toward the Atg32/Atg11 proteins have not yet been identified. 

## 8. Factors Modulating Mitophagy

In addition to the Atg32 protein, several other diverse proteins have been identified as pivotal for mitophagy, but indispensable for other forms of autophagy, suggesting that mitochondrial elimination can be facilitated and controlled by multiple specific mechanisms. In this light, several signaling pathways might be involved or participate in mitophagy following its induction. For example, Mendl et al. (2011) showed the Whi2 protein, which is involved in regulating several fundamental cell pathways such as general stress response, cell cycle arrest, and the Ras–protein kinase A (PKA) signaling pathway, to be required for efficient mitophagy induction by rapamycin treatment [75]. Results from another lab showed that Whi2p is dispensable for mitophagy induction by nitrogen starvation [76]. Protein Whi2 inhibits the TORC1 pathway and could regulate mitophagy via nutrient-signaling pathways that employ stress response transcription factors, such as Msn2 or Ras–PKA [77]. 

Similarly, proteins Atg33 [46] and Aup1 [43] play a role in mitophagy during the stationary phase of growth, but are not essential for mitophagy induced during nitrogen starvation.

Another example is the Uth1 protein, which is thought to be involved in micromitophagy, but is not required for macromitophagy [39].

It has been shown that the Isc1 protein, a sphingolipid phospholipase involved in ceramide synthesis, regulates mitophagy and mitochondrial dynamics [78]. In this study, the authors observe a hyperactivation of mitophagy in the *isc1*Δ mutant associated with Dnm1p-dependent mitochondria fission. 

Concerning the link between mitochondrial dynamics and mitophagy, the observations are controversial. Mitochondria are highly dynamic organelles with constant fusion and fission, and fission is often regarded as a prerequisite for mitophagy. In yeast, among all the proteins involved in fission, only Dnm1p is required for mitophagy [46,76], but opposite results were found by two teams, Mendl et al. (2011) and Yamashita et al. (2016) [75,79].

Recently, Vigié et al. (2019) showed that the mitochondrial PE pool, which involves the activity of mitochondrial phosphatidyl decarboxylase (Psd1), is required for mitophagy induced by nitrogen deficiency. In contrast, in the stationary phase of growth, the activity of non-mitochondrial phosphatidyl decarboxylase 2 (Psd2) is required for mitophagy [68].

These observations are comparable to what happens in *K. phaffii* during the process of lipophagy. Kumar et al. (2020) suggested a unique molecular requirement of the stationary-phase-induced lipophagy versus nitrogen starvation-induced lipophagy. The *prl1*Δ mutant is deficient for the lipophagy induced during the stationary phase, and not by starvation [80].

## 9. Physiological Role of Mitophagy in *S. cerevisiae*

Although the first electron microscope images revealing the presence of mitochondria in lysosomes of mammalian cells date back to 1957 [81], and similar pictures were captured in yeast vacuoles 30 years ago [33], the physiological relevance of selective mitochondrial elimination remained unclear until recently. In mammalian cells, the regulation of the quantity and quality of mitochondria is a crucial task because these organelles are sources of both necessary energy and the harmful ROS, which are the main triggers of the alteration of biological structures.

The crucial contribution of autophagy to mitochondrial homeostasis maintenance, by eliminating the altered mitochondria in mammalian cells, suggests that mitophagy can also play an important physiological role in unicellular organisms, such as yeast. Studies investigating the physiological role of mitophagy in yeast focus mainly on examining the phenotypes associated with mitophagy-deficient *atg32*Δ and *atg11*Δ cells. When wild-type cells in respiratory growth encounter nitrogen starvation, they initiate mitophagy and quickly eliminate the excess of mitochondria preferably located near the vacuoles that have proliferated during respiratory growth. As a result, ROS production from mitochondria is suppressed, and the mitochondria escape oxidative damage.

By contrast, in mitophagy-deficient cells, undegraded mitochondria become old and damaged and produce more ROS during nitrogen starvation and in the stationary phase. ROS damage mitochondria themselves, and the mitochondria produce more ROS in a vicious circle, which ultimately leads to mitochondrial DNA mutations/deletions. Moreover, yeast cells appear to have developed a mechanism that upregulates mitophagy to avoid the inheritance of mutated mtDNA [82]. Thus, mitophagy in yeast contributes to the reduction in oxidative stress and maintenance of mitochondrial morphology, and the preservation of genome stability [83,84]. This process is very important for yeast in nature because the cells frequently shift their metabolism between fermentation and respiration, and they are always at risk of starvation. 

Data published by Richard et al. (2013) also suggest that mitophagy can maintain the survival of chronologically aging yeast limited in calorie supply (CR) by sustaining a healthy population of functional mitochondria [85]. Under CR conditions, mitophagy not only defines the size and number of mitochondria, but also their shape and morphology and their ability to exist as a network. In addition, mitophagy is involved in the regulation of cellular lipid homeostasis, and it has been shown that this enables the aging process [85]. 

Recently, it was shown that mitophagy is induced during alcohol brewing [86]. The absence of Atg32p enhances the production of CO_2_ and ethanol. Fermentation is also reduced in the *atg11*Δ and *atg8*Δ mutants, which suggests that mitophagy defects specifically strengthen fermentation. In addition, mitophagy in yeast improves its tolerance to ethanol [87].

Recent data have also shed light on the physiological role of Atg32-dependent mitophagy in yeast metabolism during respiratory growth and heat-induced mitochondrial stress. This occurs by maintaining the production of S-adenosylmethionine (SAM), which converges to form spermidine leading to cytoprotective nitric oxide response during heat stress [88].

## 10. Mitophagy in Other Yeast Species

The most common yeast model for the study of mitophagy is the budding yeast *S. cerevisiae*. Recent studies also reveal the presence of similar regulatory mechanisms in other fungi. Atg32 protein is conserved in *Candida glabrata* and *Komagataella phaffii (Pichia pastoris*) but not in *Schizosaccharomyces pombe*. 

During infection with a human opportunistic pathogen *C. glabrata*, the amount of free iron ions inside the host is restricted and upregulation of Atg32p contributes to longevity under this condition [89]. Moreover, the absence of Atg32p significantly reduced the virulence of *C. glabrata*, which suggests mitophagy plays a role in survival during iron deficiency in addition to being induced during infection. 

*Kluyveromyces marxianus* is a thermotolerant, ethanol-producing yeast that requires oxygen for efficient ethanol fermentation. It has been shown that mitophagy was induced by anoxia, but not under nitrogen starvation, and Atg32 and Atg8 proteins are required for this [90].

In *P. pastoris* (*K. phaffii*), Aihara et al. (2014) identified and characterized the ScAtg32 homolog. Interestingly, they found that PpAtg32 protein was barely detectable before induction of mitophagy, but was rapidly expressed after activation of mitophagy by starvation. As ScAtg32p, PpAtg32p undergoes phosphorylation when mitophagy is induced [49].

The fission yeast *S. pombe* is evolutionarily distant from *S. cerevisiae* [91]. The identification and characterization of proteins essential for starvation-induced autophagy in *S. pombe* revealed interesting differences between the autophagic mechanisms of the two yeast models. For example, although budding yeast Atg11p is needed for all selective types of autophagy, and is indispensable for bulk autophagy, its fission yeast counterpart is also necessary for nonspecific autophagy [92]. Thus, the Atg11 requirement cannot be used as evidence to assess whether a cargo is selectively targeted by autophagy in *S. pombe*. As in *S. cerevisiae*, nitrogen starvation induced autophagic elimination of mitochondria in this fission yeast, but the molecular mechanism underlying this degradation, and whether this pathway is selective to mitochondria, remains uncertain [93,94]. In their recent work, Fukuda et al. (2020) identified the protein Atg43 as a new mitophagy receptor in *S. pombe* [95]. 

*Candida albicans* is an opportunistic pathogenic fungus commonly found in humans. It can cause severe infection in immunocompromised individuals. The *ATG* genes are highly conserved in this fungus, and autophagy can be activated by nitrogen deficiency or aging. In *C. albicans*, a protein homologous to Atg32 has not yet been discovered, but the scaffolding protein Atg11 has a more significant role in selective and nonselective autophagy than in *S. cerevisiae* [96]. Recent work highlights the role of the Mcp1 protein belonging to the vacuole and mitochondria patch (vCLAMP) complex, which is involved in the maintenance of mitochondrial function and mitophagy [97]. It was reported that the Mcp1 protein plays a role in preserving mitochondrial function and mitophagy [97]. 

The methylotrophic yeast species *Hansenula polymorpha* and *P. pastoris* were used as model organisms to study mainly pexophagy [98]. Presence of methanol in growth medium induces a massive peroxisomal biogenesis in these yeasts. In methylotrophic yeast species, peroxisomes are massively induced when cells are grown in methanol because they harbor key enzymes required for methanol metabolism. Upon a shift of methanol-grown cells to media containing glucose or ethanol, peroxisomes become redundant and are rapidly and selectively degraded. 

The role of the autophagic proteins Atg20 and Atg24 were little studied outside of *S. cerevisiae*. In *P. pastoris*, the ortholog of the ScAtg24 protein is required for pexophagy, but the function of the Atg20-related protein is unrevealed [69]. In the rice-blast fungus *Magnaporthe oryzae*, the ortholog of ScAtg24 is essential for mitophagy [99] and the Atg20-related protein is needed for pexophagy [100,101]. Mammalian homologs of ScAtg20 and ScAtg24 also exist, but their involvement in autophagy is unknown [102].

## 11. Yeast *Saccharomyces cerevisiae* Could Be Used to Express Human Proteins

The process of mitophagy is less complex in the yeast *S. cerevisiae* than in mammals due to the presence of a single pathway requiring the Atg32 receptor (see [103] for review). In contrast, mammals have the following two pathways: the ubiquitin Pink/Parkin pathway utilizing adapter protein involvement (P62, NBR1, OPTN, NDP52, TAX1), and the receptor-dependent pathway with the identification of several receptors (BNIP3, BNIP3L/NIX, FUNDC1, Bcl2-L-13, FKBP8, Ambra1 found on the outer mitochondrial membrane, and recently described inner mitochondrial membrane protein, PHB2). Yeast is a single-cell organism that does not require differentiation or maturation steps to grow. However, it remains an interesting model used to answer questions that remain unanswered.

In mammalian cells, Bcl2-L-13 has proven its involvement in mitophagy as well as mitochondrial fragmentation, and was identified as the homolog of yeast Atg32p. Bcl2-L-13 is localized in the outer mitochondrial membrane and possesses the conserved LIR domain, which allows it to interact with LC3. Heterologous expression of Bcl2-L-13 in the *atg32*Δ yeast mutant brings back the capability to activate mitophagy dependent on the presence of the LIR domain. Furthermore, the expression of Bcl2-L-13 did not repair the inability to induce mitophagy in the *atg7*Δ yeast mutant, which indicates Bcl2-L-13-mediated mitophagy uses known autophagic molecular machinery [104]. It would also be interesting to express other mitophagy receptors, such as FUNDC1 and NIX in yeast, to test if there can be complementation in the absence of Atg32p. 

The use of a simple yeast genetic model to perform large-scale screenings has provided insights into the molecular mechanisms of several human diseases [105]. For example, several yeast models have been developed to study α-synuclein toxicity and better understand disease-related defects in vesicular trafficking, oxidative stress, and mitochondrial dysfunction [106,107,108,109]. 

*S. cerevisiae* has also been used to analyze the biological function of the human Parkin protein. The expression of Parkin in yeast increases the yeast’s chronological lifespan and resistance to oxidative stress through mitochondria-dependent pathways. In addition, Parkin translocates to the mitochondria in response to H_2_O_2_, leading to further mitochondrial degradation. Parkin-induced resistance to H_2_O_2_ depends on the autophagic pathway and the mitochondrial protein Por1 [110]. This type of study can improve the understanding of Parkin’s functions and highlight its new partners.

The yeasts are also an important model for studying mevalonate kinase deficiency (MKD), an autosomal recessive disease that causes systemic autoinflammatory problems in humans. To understand the effects of mevalonate kinase (MKV) gene impairment in the absence of animal or cell models, the authors used in their study the *erg12-d* mutant strain S. *cerevisiae* (orthologous of MKV) that maintains only 10% of mevalonate kinase activity. With this model, they show that MKD causes mitochondrial dysfunction, which can ultimately lead to autophagy/mitophagy induction. These processes are not completed; therefore, damaged mitochondria may not be recycled and may generate cellular metabolic dysfunction. Understanding this mechanism may help researchers decipher the nonspecific autoinflammatory response observed in patients with MKD [111,112].

## 12. Future Directions

How are mitochondria targeted for degradation? One of the roles of mitophagy is the removal of damaged mitochondria. From what level of damage is the process triggered? Unlike in mammalian cells, using a decoupler, such as CCCP, or a respiratory chain inhibitor, like Antimycin A, does not induce mitophagy in yeast. The process of mitophagy to eliminate damaged mitochondria is established in yeast during the stationary phase, in conditions that allow the accumulation of oxidized proteins and lipids (Figure 2, right panel). In contrast, when yeast cells are subjected to nitrogen starvation in the exponential phase of growth, mainly undamaged mitochondria are degraded by mitophagy, providing nutrients to the yeasts (Figure 2, left panel). In both scenarios, the presence of Atg32p is required. Atg32p is constantly present in the outer membrane of the mitochondria and must be phosphorylated by casein kinase 2 (CK2) for mitophagy to be induced [50]. At present, Atg11 and Atg8 are the only two proteins identified that interact with Atg32p. Ppg1, the PP2A-like protein phosphatase, allows Atg32 dephosphorylation [56,113]. Upon mitophagy initiation, Far8, one of the core proteins of the FAR complex, dissociates from Atg32 on the mitochondria’s surface, facilitating CK2-mediated Atg32 phosphorylation, stabilizing Atg32–Atg11 interactions, and promoting mitophagy. Despite the data, the signal that induces the dephosphorylation or the phosphorylation of Atg32 is not known. Different data reveal that this signal would not have to be the same in the starvation and the stationary phase of growth because mitophagy does not play the same role. It would be interesting to investigate whether other proteins could interact with Atg32 under the different conditions of mitophagy induction to clarify the different stages of the process.

It was also observed that the source of phosphatidylethanolamine (PE), a phospholipid essential for the autophagic machinery, was not the same, depending on mitophagy induction. In nitrogen deficiency conditions, it is the mitochondrial PE pool that is required, and it has been observed that only the mitochondria present in the vacuole’s vicinity is degraded. Under stationary growth phase conditions, the origin of PE is more diverse [68]. The Atg32 protein undergoes other post-translational modifications not yet identified [60]. In addition, Okamoto’s lab reported that N-terminal acetyltransferase A (NatA), an enzymatic complex composed of the catalytic subunit Ard1 and the adaptor subunit Nat1, is crucial for mitophagy in yeast because mitophagy is strongly suppressed in mutant cells that lack *ARD1*, *NAT1*, or both genes [51]. Further, Atg32 induction was partially suppressed in cells that lacked NatA. The N-acetylation targets required for mitophagy have not yet been identified.

Moreover, it was demonstrated that Atg32 phosphorylation, which is required for mitophagy facilitation, is altered in the breathing *natA*Δ mutant [53]. It has also been shown that the Atg32 protein was ubiquitously degraded by the proteasome in the stationary growth phase, which controls the mitophagy level [59]. This regulation by phosphorylation and ubiquitination is also reported for the mitophagy receptor FUNDC1 [114,115,116,117]. 

**Figure 3:**
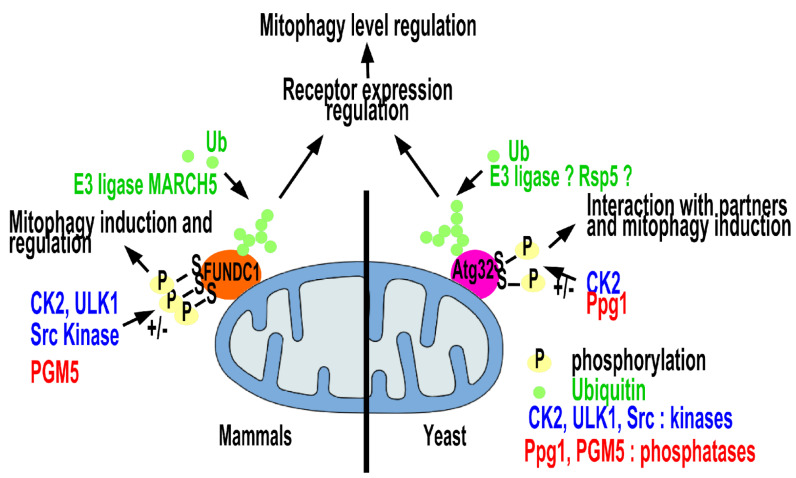
Regulation of receptor-involved mitophagy in yeast and mammals. Following the mitophagy induction, both yeast Atg32 and mammalian FUNDC1 receptors undergo the same type of post-translational modifications. Phosphorylation of mitophagic receptors plays a pivotal role in either promoting or inhibiting mitophagy depending on the circumstances. In reaction to hypoxia or depolarization of mitochondrial membrane potential, the mitochondrial phosphatase PGAM5 interacts with FUNDC1 and dephosphorylates the serine 13 residue. This enhances its interaction with LC3, which ultimately leads to mitophagy induction. The kinase CK2 phosphorylates FUNDC1 to reverse the effect of PGAM5 in mitophagy induction. In addition, the mitochondrial E3 ligase MARCH5 plays a role in regulating hypoxia-induced mitophagy by ubiquitinilating FUNDC1 and causing its degradation to limit excessive mitochondrial degradation. In yeast, during nitrogen starvation or in stationary phase, CK2 kinase phosphorylates Atg32 protein on serines 114 and 119; these modifications are essential for the initiation of mitophagy. Ubiquitination is also involved in regulation of Atg32 activity.

It was observed that upon hypoxia or loss of mitochondrial membrane potential, the FUNDC1 receptor interacts with the mitochondrial phosphatase PGAM5, which catalyzes the removal of the phosphate group from serine at position 13. This enhances its interactions with LC3. The CK2 kinase phosphorylates FUNDC1 to reverse the effect of PGAM5 in activating mitophagy. In addition, the mitochondrial E3 ligase MARCH5 is involved in regulating hypoxia-induced mitophagy by ubiquitinating FUNDC1, leading to its degradation and limiting excessive mitochondrial degradation [116] [Figure 3].

Studies have shown that this BNIP3L receptor dimerizes in the outer membrane of the mitochondria because of the presence of the GxxxG motif in the transmembrane domain [118]. The GxxxG motif may be involved in high-affinity association of transmembrane helices [119]. Recently, Marinković et al. (2021) examined the mechanisms of BNIP3L/NIX-mediated mitophagy, the pathway responsible for the degradation of normal, healthy mitochondria during the terminal differentiation (programmed mitophagy) of reticulocytes [120]. The authors proposed BNIP3L/NIX dimerization involving the GxxxG motif in the transmembrane domain as a potentially novel molecular mechanism underlying BNIP3L/NIX-dependent mitophagy. This dimerization allows for a more robust recruitment of autophagosomes and a progression of mitophagy. Furthermore, Marinković et al.’s results suggested that the combined mechanism of phosphorylation and receptor dimerization is needed for proper BNIP3L-dependent mitophagy initiation and progression. The presence of this type of motif is found also in the transmembrane domains of other mitophagy receptors directing dysfunctional mitochondria to the autophagosomes, such as FUNDC1 (motif GFVGG) and Atg32 (motif GISFG). This raises the questions of whether these GxxxG motifs allow the dimerization of these receptors, thus affecting the course of mitophagy. 

Another important question concerns the role of the endoplasmic reticulum (ER) and MAMs in mitophagy. It has been shown that the loss of mitochondrial–ER tethering is responsible for mitophagy defects in *mdm10*Δ, *mdm34*Δ, and *mmm*1Δ ERMES mutants [121]. It was proposed that these contacts would make it possible to bring membranes derived from the ER to form the mitophagosome [115]. Another study showed that the ubiquitination of Mdm34 and Mdm12 by the E3 ligase Rsp5 is required for efficient mitophagy [122]. More recently, it has been shown that mitophagy during prolonged respiratory growth is strongly impaired in yeast cells lacking Get1/2, a transmembrane complex mediating the insertion of tail-anchored (TA) proteins into the ER membrane [123]. The connection between ER and mitochondria also could be made through this Get1/2 complex and the complex FAR that Dr. Kanki’s team highlighted [56,112]. We must now understand how these two complexes interact and the mechanisms involved in regulating mitophagy through the key protein Atg32.

## 13. Conclusions

The yeast *S. cerevisiae* has demonstrated selective degradation of mitochondria by mitophagy, thus providing a useful model to understand the molecular mechanism, actors, and regulation of this process. This model can help answer unresolved questions, and the study of mitophagy in yeast can help answer the following three important questions: First, the molecular mechanisms of selecting damaged mitochondria from healthy ones are still highly obscure; how are the mitochondria that must be degraded recognized? The presence of the phosphorylated or dephosphorylated Atg32 receptor alone should not be sufficient. Second, is the interplay between phosphorylation of Atg32 and other posttranslational modification needed for proper mitophagy? Third, MAMs—the regions of the ER that mediate communication between the ER and mitochondria—have been demonstrated to be involved in autophagy; what are the relationships between MAMs and mitophagy? What proteins mediate the involvement of MAMs in the expansion of autophagosomes around mitochondria designed for degradation? 

With the development of innovative cellular and molecular technologies implemented to study mitophagy in more evolved organisms (such as mammals) in which mitophagy plays a more important role in the quality control of mitochondria than in yeast—and in which dysfunctions of this process are involved in many pathologies—key information about important potential targets for the treatment of diseases related to mitophagy is expected to be obtained.

## Data Availability

Data sharing not applicable.

## Abbreviations

AMBRA1	autophagy and beclin 1 regulator 1
BNIP3	BCL2 interacting protein 3
BNIP3L/NIX	BCL2 interacting protein 3 like
CCCP	Carbonyl cyanide *m*-chlorophénylhydrazone
CK2	casein kinase 2
DHFR	Dihydrofolate reductase
FCCP	Carbonyl cyanide 4-(trifluoromethoxy)phenylhydrazone
FKBP8	FKBP prolyl isomerase 8
FUNDC1	FUN14 domain containing 1
GFP	green fluorescent protein
GSH	reduced glutathione
LIR	LC3-interacting region
MAM	mitochondria associated membrane
NAC	N-acetyl cysteine
PHB2	prohibitin 2
PINK1	PTEN induced kinase 1
RFP	red fluorescent protein
